# Early Cretaceous angiosperms and beetle evolution

**DOI:** 10.3389/fpls.2013.00360

**Published:** 2013-09-12

**Authors:** Bo Wang, Haichun Zhang, Edmund A. Jarzembowski

**Affiliations:** ^1^Steinmann Institute, University of BonnBonn, Germany; ^2^State Key Laboratory of Palaeobiology and Stratigraphy, Nanjing Institute of Geology and Palaeontology, Chinese Academy of SciencesNanjing, China; ^3^Department of Earth Sciences, The Natural History MuseumLondon, UK

**Keywords:** beetle, angiosperm, Cretaceous, pollinator, fossil, coevolution

## Abstract

The Coleoptera (beetles) constitute almost one–fourth of all known life-forms on earth. They are also among the most important pollinators of flowering plants, especially basal angiosperms. Beetle fossils are abundant, almost spanning the entire Early Cretaceous, and thus provide important clues to explore the co-evolutionary processes between beetles and angiosperms. We review the fossil record of some Early Cretaceous polyphagan beetles including Tenebrionoidea, Scarabaeoidea, Curculionoidea, and Chrysomeloidea. Both the fossil record and molecular analyses reveal that these four groups had already diversified during or before the Early Cretaceous, clearly before the initial rise of angiosperms to widespread floristic dominance. These four beetle groups are important pollinators of basal angiosperms today, suggesting that their ecological association with angiosperms probably formed as early as in the Early Cretaceous. With the description of additional well-preserved fossils and improvements in phylogenetic analyses, our knowledge of Mesozoic beetle–angiosperm mutualisms will greatly increase during the near future.

## INTRODUCTION

The Coleoptera (beetles) constitute almost one–fourth of all known life-forms on earth, and their extraordinary species richness is probably due to the elevated survival of lineages and their sustained diversification in a variety of niches (Hunt et al., 2007; Ikeda et al., 2012). Beetles are among the most important pollinators of flowering plants, especially basal angiosperms (Thien et al., 2009), and they are also thought to be among the earliest insect visitors and pollinators of angiosperms (Bernhardt, 2000). The diversification of some of the most species rich extant lineages of beetles may have been driven by co-radiations with angiosperms (Farrell, 1998). In addition, the early partnerships between angiosperms and pollinating insects were also major factors in the currently recognized radiation of the angiosperms (Hu et al., 2008).

The Early Cretaceous is a time of important developments in angiosperms (Friis et al., 2010). The earliest unequivocal remains of angiosperms are generally thought to be pollen grains in the early Hauterivian (~130 Ma; Friis et al., 2010), despite claims based on other fossils and molecular analyses (Bell et al., 2010; Wang, 2010; Smith et al., 2011). The fossil record shows that angiosperms rose to dominance during the Albian–Cenomanian (112–93.6 Ma), and become forest dominants during the Campanian–Maastrichtian (84–65.5 Ma; Peralta-Medina and Falcon-Lang, 2012). The angiosperm radiations provided new food resources and habitats, and had a profound effect on beetles and other insects.

The Early Cretaceous is also an important period for the evolution of beetles, including the decline of archostematan beetles, ecological success of adephagan beetles, and the first appearance and divergence of some major polyphagan lineages (Ponomarenko, 2002). The Scarabaeoidea, Tenebrionoidea, Curculionoidea, and Chrysomeloidea are among the commonest pollinators of most extant basal angiosperms (Bernhardt, 2000; Thien et al., 2009). The probable impact of floristic changes on beetles during the Early Cretaceous has been widely accepted, but supporting fossils are still very few (Labandeira, 1998; Grimaldi, 1999; Labandeira and Currano, 2013). Recently abundant Early Cretaceous fossils have been described and our knowledge of the evolution of beetles has improved greatly. These new fossils, therefore, provide important clues to explore the co-evolutionary process between beetles and angiosperms. In this paper, we review the fossil record and early evolution of these four Early Cretaceous polyphagan groups, and briefly discuss their probable ecological associations with early angiosperms.

## FOSSIL RECORD

### SCARABAEOIDEA

Scarabaeoidea (scarab beetles) is a cosmopolitan monophyletic superfamily comprising around 35,000 described species (Scholtz and Grebennikov, 2005). They are commonly stout-bodied beetles, sometimes with bright metallic colors, measuring between 1.5 and 160 mm long (Bai et al., 2012). Their adult fossils can be distinguished by the following features: distinctive, clubbed antennae; front legs broad and adapted for digging; and meso- and metatibia commonly with transverse, setose, or spinose keels on an outer surface (Krell, 2006). The earliest unambiguous fossils, represented by several families, are recorded from the Middle Jurassic of Daohugou, China and the Upper Jurassic of Karatau, Kazakhstan (Nikolajev et al., 2011; Bai et al., 2012). The fossil record of Scarabaeoidea has been reviewed by Krell (2006) and Nikolajev (2007), and the biodiversity pattern in terms of species numbers from the Cenozoic and Mesozoic eras is summarized by Bai et al. (2012). The overwhelming majority of fossil scarabaeoids have been discovered from the Early Cretaceous of China, Russia, and Brazil, most of which require detailed descriptions (Krell, 2006; Zhang et al., 2010). A recent molecular analysis suggests that most scarab families may have originated in the Cretaceous (McKenna and Farrell, 2009). However, the discovery of scarab fossils from the Middle to Late Jurassic indicate that they are much older than their estimated divergence times calculated from previous molecular evidence (Nikolajev et al., 2011; Bai et al., 2012), and most of which belong to families that have been found in the Early Cretaceous (Krell, 2006; Nikolajev, 2007). Consequently, most families of Scarabaeoidea originated significantly before the mid-Cretaceous angiosperm radiation, probably during the Middle-Late Jurassic.

### TENEBRIONOIDEA

The Tenebrionoidea (including darkling beetles) is one of the most abundant superfamilies of beetles, comprising about 35,000 described species in 30 families worldwide (Hunt et al., 2007). Adult tenebrionoids are distinguished by an aedeagus of heteromeroid condition, trochanterofemoral articulations oblique, and tarsal formulae of 5-5-4, 4-4-4, 3-4-4 or rarely 3-3-3 (Lawrence et al., 2010). The oldest definite records of this group are mordellid-like beetles from the Middle Jurassic of Daohugou, China, and several families are also described from the Upper Jurassic of Karatau, Kazakhstan (see annotated list of Mesozoic tenebrionoid beetles given by Wang and Zhang, 2011).

The phylogenetic relationships among the families of Tenebrionoidea are still poorly understood (Beutel and Friedrich, 2005; Lawrence et al., 2010). The Mordellidae are thought to be among the most basal groups of Tenebrionoidea based on molecular analysis (McKenna and Farrell, 2009). Some Early Cretaceous fossils have been attributed to the extinct subfamily “Praemordellinae” which was placed in Mordellidae on the following characters: tarsal formula 5-5-4; body wedge-shaped, elongate, and arched with fine pubescence; head deflexed, constricted behind the eyes to form a neck; and abdomen (not pygidium) extending beyond elytra (Liu et al., 2007). These characters are not autapomorphies of Mordellidae, and all can be found in the Ripiphoridae and Scraptiidae. Instead, these characters suggest Praemordellidae is a stem group which probably includes ancestors of Mordellidae and of other groups, such as Ripiphoridae. The earliest definitive Mordellidae are reported from the earliest Cenomanian (Late Cretaceous) Burmese amber (Grimaldi et al., 2002; Shi et al., 2012). The adults of modern Mordellidae are phytophagous, apparently feeding on the pollen of many plants, especially of umbellifers (Apiaceae) and composites (Asteraceae; Jackman and Lu, 2002). In the Cretaceous, fossils appear relatively diverse and show that several advanced tenebrionoids, such as Rhipiphoridae and Tenebrioninae, definitely occur during this interval (e.g., Perrichot et al., 2004; Liu et al., 2007; Kirejtshuk et al., 2012). Both fossils and molecular analysis suggest that most families of Tenebrionoidea originated during or before the Early Cretaceous.

### CURCULIONOIDEA

The Phytophaga represents the largest radiation of beetles (Coleoptera), and is the second most species rich lineage of plant-feeding animals after the Lepidoptera (Grimaldi and Engel, 2005). It is a well-defined monophyletic group supported by both morphological and molecular data, comprising the reciprocally monophyletic groups Curculionoidea (weevils) and Chrysomeloidea (longhorn and leaf beetles; Marvaldi et al., 2009).

The Curculionoidea (weevils), containing about 62,000 species within 5,800 described genera, are the most diverse group of beetles (Oberprieler et al., 2007). They are easily recognizable by a rostrum or snout projecting forward and downward from the head, with mandibles or jaws positioned at the rostral tip (Thompson, 1992). Weevils are essentially phytophagous beetles, and some are serious pests of agricultural crops and stored food products. Their fossil record is quite extensive and constitutes the dominant group among the Phytophaga in the Mesozoic fossil record (Arnoldi et al., 1977). The oldest weevils, belonging to the Nemonychidae, occur in the Middle Jurassic of Daohugou, China (undescribed specimens) and Upper Jurassic of Karatau, Central Asia (Oberprieler et al., 2007). An updated list of Mesozoic fossils is provided by Soriano et al. (2006) and Legalov (2012). Abundant weevils were recorded from the Lower Cretaceous of Russia, Spain, China, and Brazil (Soriano et al., 2006; Davis et al., 2013). However, no weevil is known from the Triassic to Early Jurassic, although there are many other beetle fossils of the same age (Gratshev and Zherikhin, 2003). One lineage, the Obrieniidae, was thought to have a relationship to the Curculionidae, but now is thought to be an example of morphological convergence or parallel evolution in an unrelated group (Gratshev and Zherikhin, 2003; Oberprieler et al., 2007).

The Nemonychidae, consisting of 21 living genera and 76 known species, is a small, relict family of weevils (Kuschel, 1983; Oberprieler et al., 2007). They are the most primitive weevils, with the most plesiomorphic features of all extant lineages. These beetles are most diverse in the Australian and Neotropical regions, with fewer species occurring in the Nearctic and Palaearctic regions. The Nemonychidae are predominantly associated with conifers, especially the family Araucariaceae, while the Pinaceae provide their common hosts in the northern hemisphere. The Nemonychidae are presumed to retain the ancestral life style of weevils, including their mobile larvae living freely (ectophytically) among the sporophylls within dehiscing male conifer strobili (cones), feeding on pollen in open pollen sacs and moving between cones (Oberprieler et al., 2007).

The Anthribidae, Attelabidae, Caridae, and Curculionidae are definitely known from the Early Cretaceous (Oberprieler et al., 2007; Kirejtshuk et al., 2009; Cognato and Grimaldi, 2010; Santos et al., 2011). Estimated divergence times indicate that initial diversification of most families occurred on gymnosperms during the Jurassic, and a massive diversification even probably began in the mid-Cretaceous (McKenna et al., 2009; Jordal et al., 2011).

### CHRYSOMELOIDEA

Most chrysomelid beetles belong to two unusually diverse families: Chrysomelidae (leaf beetles) with more than 35,000 described species, and Cerambycidae (longhorn beetles) with over 20,000 described species (Marvaldi et al., 2009). The Chrysomelidae are typically small to medium-size beetles with an elongate-oval body form, and adults that commonly consume leaves or floral elements (Riley et al., 2002). Cerambycidae are generally large insects with raised antennal insertions and long antennae (Turnbow and Thomas, 2002). Adult Cerambycidae feed on leaves, meristematic cambial tissue or pollen, and their larvae generally mine the phloem of trees or bore into heartwood (Hanks, 1999). Almost all species of extant Chrysomeloidea are phytophagous, and most phytophagous Chrysomeloidea feed on angiosperms (Farrell, 1998). Their great diversity has been commonly attributed to co-radiation with early angiosperms (Farrell, 1998; Wilf et al., 2000; Farrell and Sequeira, 2004; but see Reid, 2000).

The fossil record of Mesozoic Chrysomeloidea is extremely poor (Grimaldi and Engel, 2005), and is reviewed recently by Wang et al. (2013). Several Late Jurassic fossils from Karatau, Kazakhstan probably are attributed to the Chrysomeloidea, but require further investigation as to their systematic position. Besides these Jurassic fossils, only two fossils of Early Cretaceous Chrysomeloidea are known. The earliest known Cerambycidae (Prioninae) and Chrysomelidae (Bruchinae) are from the Early Cretaceous of China and the Late Cretaceous Canadian amber respectively (Poinar, 2005; Wang et al., 2013). Estimated divergence times suggest that most modern families of Chrysomeloidea originated during the Jurassic period (Farrell, 1998; McKenna and Farrell, 2006, 2009; Hunt et al., 2007; Wang et al., 2013). Chrysomelidae may occur in the Middle Jurassic (Wang et al., 2013), and all major subfamilies of Chrysomelidae originated before the beginning of the Albian (Kergoat et al., 2011; Wang et al., 2013).

## PROBABLE BEETLE–ANGIOSPERM ASSOCIATIONS

Mutualisms between fossil insects and plants are among the most interesting biological associations. The most compelling evidence of early interactions between insects and the reproductive organs of plants is in the form of pollen preserved in the coprolites or guts of fossil insects (Bronstein et al., 2006; Labandeira et al., 2007). The earliest records of pollen consumption are from permineralized coprolites of medullosan seed-fern pollen and tree-fern spores from the Late Pennsylvanian Calhoun Coal (Labandeira, 2006), and from generalized polyneopterans from the Permian with gymnosperm pollen preserved in their guts (Rasnitsyn and Krassilov, 1996). Although some pollen grains were found in the guts of several Mesozoic groups, such as wasps and crickets, no record is reported from Mesozoic beetles. The preservation of gut contents appears unrelated to the preservational quality of the insects. For example, no gut contents hitherto have been found in the well-preserved Jurassic Daohugou specimens. Further investigation of additional insects from various Mesozoic fossil localities may reveal direct evidence.

Other direct evidence for the evolution of insect pollination is the preservation in fossil angiosperm flowers of specialized structures that attract favored insect partners and promote pollen delivery (e.g., Hartkopf-Froder et al., 2012). The Early Cretaceous evidence is, however, very scarce due to the rarity of well-preserved three-dimensional flowers. So far, only Gandolfo et al. (2004) offer a compelling argument that the pollination of members of the Nymphaeaceae by beetles represents an ancient mutualistic partnership which can be traced back to the mid-Cretaceous.

Indirect evidence include the structure of the pollen-collecting mouthparts of beetles. Modern flower-visiting beetles often possess conspicuous eyes and combs of setae on the mandibles, palps or other mouthparts, for example in the Oedemeridae and Mordellidae (Barth, 1985; Krenn et al., 2005). Bernhardt (1996) reviews the three different mechanisms that beetles may employ to digest the contents of pollen grains. Some structures of fossil mouthparts clearly are visible in amber-entombed beetles (e.g., Kirejtshuk et al., 2009) as well as occasional specimens from well-preserved compressions (e.g., Wang et al., 2012). Further examination of the mouthparts from some probable Mesozoic pollen-feeding beetles is needed.

Other indirect evidence consists of observations on pollination systems of the most basal angiosperms, including the ANITA group and Magnoliidae. Nine families, consisting of Scarabaeidae; Mordellidae, Oedemeridae, and Scraptiidae within Tenebrionoidea; Cerambycidae and Chrysomelidae within Chrysomeloidea; Curculionidae; Nitidulidae; Staphylinidae, are pollen vectors of specialized flowers produced by magnoliids. Two families, Scarabaeidae and Chrysomelidae, are pollinators of the Nymphaeaceae (Bernhardt, 2000; Bernhardt et al., 2003). Ervik and Knudsen (2003) have also presented evidence that members of the Nymphaeaceae are pollinated by scarabaeid beetles. As discussed above, at least four lineages of beetle pollinators of extant basal angiosperms occurred minimally by the Early Cretaceous, suggesting that their ecological association with angiosperms probably appeared at the same time or earlier with gymnospermous groups and even ferns. Their phytophagous-adapted structures, including herbivory or pollination, were probably used first on a variety of gymnospermous groups and even ferns (Labandeira et al., 2007). A similar condition is present in other insects such as scorpionflies (Ren et al., 2009) and thrips (Peñalver et al., 2012). The beetle adaptation on gymnospermous groups before the rise of angiosperms is beyond the scope of this paper, and will be discussed in the following paper.

Phylogenetic analysis based on fossil, morphological and molecular data is a powerful tool for investigating the evolution of beetle–angiosperm interactions (McKenna, 2011). Detailed investigations of the Tenebrionoidea and Scarabaeoidea are lacking, but some progress has been made on the Phytophaga. Molecular analysis of the Curculionoidea indicates that their diversification into most family level lineages took place during the Jurassic and a subsequent radiation closely followed the currently recognized rise of the angiosperms (McKenna et al., 2009; but see Franz and Engel, 2010). A similar scenario has also been proposed for the curculionoid sister group, Chrysomeloidea (Wang et al., 2013). Initial diversification of the Chrysomeloidea most probably occurred on gymnosperms in the mid-Mesozoic, when conifers and other gymnospermous plants dominated forest and other ecosystems such as open-canopied woodland, and perhaps some wetlands (Wang et al., 2013). The diversification of the angiosperm-associated subfamilies widely corresponds with the rapid increase of angiosperm productivity and the rise of angiosperm-dominated forests which may have provided new, diversified ecological niches as well as abundant food, and facilitated the radiation of the Chrysomelidae (Wang et al., 2013). Consequently, the Chrysomeloidea and Curculionoidea may have a similar evolutionary trajectory: both show high lineage survival rates and the rise of the angiosperms contributed to their modern high diversity. Nevertheless, because of uncertainties about higher-level relationships and divergence times in these beetle lineages, the detailed evolutionary history of beetle–angiosperm interactions remains unclear.

## CONCLUSION

Scarabaeoidea, Tenebrionoidea, Curculionoidea, and Chrysomeloidea currently are among the common pollinators of the most basal angiosperms. Both the fossil record and molecular analyses reveal that these four groups had already diversified during or before the Early Cretaceous. Their divergence is clearly before the currently recognized radiation of angiosperms and achieved widespread floristic dominance, suggesting that their ecological association with angiosperms probably formed as early as the Lower Cretaceous. In addition to small beetles, other common pollinator groups were around at this time pollinating basal (ANITA-grade) angiosperms, such as thrips, nematoceran flies, moths, scorpionflies, and small parasitoid wasps (Labandeira, 2005; Labandeira and Currano, 2013). In this review we have only scratched the surface of the evolution of Early Cretaceous beetle–angiosperm mutualisms. However, the Early Cretaceous associations between beetles and angiosperms are far from completely known. Thanks to the discovery of better preserved fossils and improvements in phylogenetic analysis, our knowledge of Mesozoic beetle–angiosperm mutualisms should greatly expanded in the immediate future.

## Conflict of Interest Statement

The authors declare that the research was conducted in the absence of any commercial or financial relationships that could be construed as a potential conflict of interest.
